# Central nervous system involvement in acute lymphoblastic leukemia: pathogenesis and targeted therapy

**DOI:** 10.1038/s41375-026-03060-8

**Published:** 2026-07-17

**Authors:** Luke Quinlan, David Yeung, Susan Heatley, Deborah White, Elyse Page

**Affiliations:** 1 Blood Cancer Program, Precision Cancer Medicine Theme, South Australian Health & Medical Research Institute (SAHMRI), Adelaide, SA Australia; 2 Adelaide University, Adelaide, SA Australia; 3 Royal Adelaide Hospital, Adelaide, SA Australia; 4 Australasian Leukaemia and Lymphoma Group (ALLG), Melbourne, VIC Australia; 5 Australian and New Zealand Children’s Haematology Oncology Group (ANZCHOG), Clayton, VIC Australia

**Keywords:** Acute lymphocytic leukaemia, Cancer microenvironment

## Abstract

Involvement of the central nervous system (CNS) is an adverse complication of acute lymphoblastic leukemia (ALL) associated with poor patient outcomes. Clinical challenges in the diagnosis and treatment of ALL in the CNS arise from an incomplete understanding of CNS disease pathogenesis, a significant barrier to developing novel therapeutics and biomarkers to address these challenges. A concerted research effort over recent years has significantly improved our understanding of CNS disease pathogenesis in ALL. Numerous CNS-infiltrating mechanisms by ALL cells have been uncovered, so recent research focuses on how ALL persists in this site. The role of the CNS microenvironment in the survival and therapy resistance of ALL is emerging, driven by ALL cell adhesion and metabolic reprogramming, revealing pathways with therapeutic potential. Here, we integrate recent advances in CNS infiltration and persistence in ALL, focusing on the signaling and metabolic frameworks that enable leukemic survival and therapy resistance once established in this site. We also evaluate novel therapeutic avenues and emerging targeted therapies that could enhance the efficacy of CNS-directed therapy in ALL.

## Introduction

Central nervous system (CNS) involvement remains a significant clinical challenge in the treatment of acute lymphoblastic leukemia (ALL). Although seldom observed at diagnosis, overt CNS involvement comprises 31% of pediatric and 5% of adult ALL cases at relapse [1–4]. Unfortunately, few clinical characteristics of ALL have been associated with CNS disease, including high-risk genomic abnormalities, hyperleukocytosis, and a T-cell phenotype [1, 5].

The diagnosis of ALL in the CNS is challenging. Gadolinium-enhanced MRI is the preferred imaging technique for diagnosing CNS involvement; however, its poor sensitivity limits its use as a standalone diagnostic tool in ALL [6]. Instead, cerebrospinal fluid (CSF) of ALL patients is routinely collected by a lumbar puncture, which is an invasive procedure, and examined by microscopic analysis (CSF cytology) [7, 8]. Flow cytometric analysis of CSF is an emerging technique that demonstrates greater sensitivity than cytology, but it has not been widely adopted in routine clinical practice [9]. Consequently, CSF cytology remains the gold standard for the diagnosis of CNS involvement. Outcomes from CSF cytology class patients into “CNS stages” which are not standardised and vary between studies, but generally follow: CNS1 ≤ 5 white blood cells/μL (non-leukemic), CNS2 ≤ 5 white blood cells/μL (leukemic), and CNS3 > 5 white blood cells/μL (leukemic) [10–12]. Technical difficulties can result in a traumatic lumbar puncture, contaminating CSF specimens with ALL cells, which can produce false-positive results and theoretically risk seeding leukemic cells into the CNS of patients [8].

CSF cytology has low sensitivity and poor reproducibility [11–13]. While the implications for CNS3 status are clear, CNS2 is not reliably reproducible and inconsistently prognostic, and CNS1 underestimates disease burden in most patients [13–15]. Before the universal incorporation of CNS-directed therapy, most ALL patients would relapse in the CNS, yet less than 5% were classified as CNS3 at diagnosis [16–18]. More recently, the study by Winick et al. found that the presence of CSF blasts, irrespective of count, is an independent predictor of poor outcomes [14]. These observations suggest current staging systems stratify overt CNS disease but underestimate low-level CNS involvement, restricting the implementation of risk-adapted therapy. More reliable and sensitive techniques for CNS staging are urgently needed.

Efforts to detect minimal residual disease in the CNS have been hindered by the invasive nature of CSF sampling, limited CSF sample volumes for analysis, and the absence of blood-based markers for CNS involvement. Recently, low-input molecular platforms to measure CNS involvement have emerged. Bespoke PCR-based assays, designed to detect structural variants in cell-free DNA, may be applicable even in CSF from samples with insufficient cell counts for cytologic analysis [19, 20]. Digital PCR and quantitative RT-PCR methods can also detect quantifiable microRNAs and mRNAs with high sensitivity in CSF from ALL patients [21, 22]. While promising, these studies are early in development and require clinical validation and integration. Until CNS disease burden and risk of CNS relapse can be accurately measured, universal CNS-directed therapy is of critical importance^15-17^.

CNS-directed therapy refers to all therapeutic interventions used to prevent and/or eradicate ALL cells within the CNS, including both systemic and intrathecal treatments. Contemporary chemotherapy regimens rely on combining agents with synergistic or additive anti-leukemic effects, while minimising overlapping toxicity. Some agents, such as vinca alkaloids and anthracyclines, penetrate the CNS poorly; others, including antimetabolites like methotrexate and cytarabine, more reliably penetrate the CNS when at high plasma concentrations [23–26]. Corticosteroids like dexamethasone and hydrocortisone have anti-leukaemic activity in the CNS to a varying extent, while asparaginase can modulate CNS disease through systemic asparagine depletion, effectively reducing CSF asparagine, despite an inability to directly penetrate the CNS itself [23, 24, 27, 28]. Some chemotherapeutics, such as methotrexate and cytarabine, are safe to use intrathecally and are routinely instilled into the CSF with or without steroids to directly increase CSF drug levels, see Table 1 [26, 29].

**Table 1:** Overview of current and emerging drugs for CNS disease in ALL.

ALL clinical development	Therapeutic name	Targets	Mechanism of action	CSF to plasma ratio (%)	Regulatory status	Disease	Trial number/Study	Outcome
In clinical use	methotrexate	DHFR	Inhibits DHFR, blocking folate-dependent nucleotide synthesis	3 [29]	Approved	ALL	NCT01117441	*n* = 868; 1-1.99 years, 8 mg; 2–2.99 years, 10 mg; 3–8.99 years, 12 mg; ≥9 years, 15 mg; overall survival = 96.3% [24]
	cytarabine	dCTP competitor	Cytidine analog inhibiting DNA polymerase	10–25 [29]	Approved	ALL	NCT01117441	*n* = 866; 1–1.99 years, 16 mg; 2–2.99 years, 20 mg; 3–8.99 years, 24 mg; ≥9 years, 30 mg; over all survival = 96.7% [24]
	hydrocortisone	NR3C1	Activates glucocorticoid receptor signalling	3–4^b^ [168]	Approved	ALL	NCT01117441	*n* = 866; 1–1.99 years, 8 mg; 2–2.99 years, 10 mg; 3–8.99 years, 12 mg; ≥9 years, 15 mg; overall survival = 96.7% [24]
	dexamethasone	NR3C1	Activates glucocorticoid receptor signalling	15 [29]	Approved	ALL	CCG-1922	*n* = 530; 6–10 mg/m2/day, overall survival = 93% [23]
	dasatinib	Abl, Src, c-Kit	Inhibits Abl, Src and c-Kit kinases	1–5 [169]	Approved	ALL	NCT00720109	*n* = 60; 60 mg/m2/day, overall survival = 86% [170]
Pre-clinical	selumetinib	MEK1/2	Inhibits MEK1/2 signalling	0.78–1.4^a^ [171]	Phase I/II	ALL	NCT03705507	*n* = 12, ≥18 years 75 mg/twice daily; ≤20 mg/m2/twice daily; complete response = 44% [172]
	GS-649443	PI3Kδ	Inhibits PI3Kδ signalling	1.8^b^ [39]	-	ALL	Yao et al.	*n* = 6 mice, 2 mg/kg, 66.7–83.3% decrease in CNS disease [39]
	copanlisib	PI3Kα/δ	Inhibits PI3Kα/δ signalling	-	Early Phase I	ALL	Ridge et al.	*n* = 4 mice, 14 mg/kg, 66.7% decrease in CNS disease [76]
	mavorixafor	CXCR4	Antagonises CXCR4 signalling	-	-	ALL	Jonart et al.	*n* = 5, 5 mg/kg, >95% reduction in CNS disease burden [47]
	AMG-487	CXCR3	Antagonises CXCR3 signalling	-	-	ALL	Gomez et al.	*n* = 8, 5 mg/kg, 50% decrease in CNS disease [79]
	bevacuzimab	VEGFA	Neutralises VEGF-A	16 [173]	-	ALL	Münch et al.	*n* = 18, 10 mg/kg, Signficant reduction in CNS disease burden in 3/4 xenograft samples [97]
	Me6TREN	N/A	Disrupts CXCL12-CXCR4 signalling	-	-	ALL	Jonart et al.	*n* = 4, 10 mg/kg, ~20% increase in survival [48]
	SW203668	SCD	Inhibits stearoyl-CoA desaturase	-	-	ALL	Savino et al.	*n* = 20, 20 mg/kg, signficant reduction in CNS disease burden in 2/4 xenograft samples [42]
in vitro	T-5224	c-FOS	Inhibits AP-1 transcriptional activity	-	-	ALL	Gebing et al.	Signficant reduction in migration to cerebral organoids [136]
Unexplored	mirdametinib	MEK1/2	Inhibits MEK1/2 signalling	1.3–1.6^a^ [147]	Phase I/II	Low-grade glioma	NCT04923126	*n* = 23, Three dose levels 2, 2.5 and 3 mg/m2/twice daily; Objective response = 63% [167]
	paxalisib	PI3K/mTOR	Inhibits PI3K–mTOR signalling	70–100^b^ [174]	Phase II/III	Glioblastoma	NCT03970447	*n* > 2300; Ongoing [175]
	abemaciclib	CDK4/6	Inhibits CDK4/6	-	Phase II	Breast cancer - brain metastasis	NCT02308020	*n* = 58; 200 mg/ twice daily; intracrainial objective response rate = 5.2% [151]
	Belzuitifan	HIF-2α	Inhibits HIF-2α transcriptional activity	-	Approved	Renal cell carcinoma - hemangioblastomas	NCT03401788	*n* = 24; 120 mg/ once daily; objective response rate = 63% [154]

Median values used where possible. The dash (-) denotes an absence of data.

^a^Data from non-human primate model.

^b^Data from murine model.

Within the CNS, repeated or prolonged exposure to chemotherapy may lead to radiculitis or neurocognitive sequelae [30, 31]. The risk is increased with concomitant radiation therapy, which may result in long-term cognitive deficits, such as impairments in attention, processing speed, fine motor functioning, and executive function [32, 33]. Acute neurological complications are also consequent of CNS-directed therapy, including seizures, headaches, visual disturbances, and cerebrovascular events [34]. Additionally, intensification of CNS-directed therapy increases the risk of life-threatening and fatal infections [35]. Modern chemotherapy regimens have significantly reduced CNS relapse compared with historical controls, but many patients still experience CNS relapse and neurocognitive sequelae, highlighting the need for improved therapeutic approaches [1, 17].

Therapeutic discovery has been stunted by an incomplete understanding of CNS disease pathogenesis in ALL, but emerging research proposes several mechanisms for further investigation. Tracking clonal distribution in murine patient-derived xenograft (PDX) models has revealed that the CNS comprises an analogous genomic composition of ALL cells to the bone marrow (BM) [36–38]. This suggests that unique neurotropic clones do not drive CNS disease; instead, most leukemic cells can infiltrate and persist in the CNS. Numerous methods of CNS infiltration by ALL cells have been elucidated in vivo using murine models, identifying distinct anatomical routes and molecular mechanisms to enter this site [36, 39, 40].

More recent studies have explored how ALL persists in the CNS. Transcriptomic analysis of CNS-derived ALL cells has identified unique transcriptional signatures of cell/matrix adhesion, metabolic reprogramming, and pro-survival signaling [38, 41–43]. Using co-culture systems of ALL cells with different cell types, isolated from the CNS, has elucidated a critical role of cell adhesion in the survival and chemoresistance of ALL in this site [44–48]. Additionally, recent studies reveal a role of metabolic stress and metabolic reprogramming in the survival and therapy resistance of ALL in the CNS microenvironment in vitro and in vivo [41–43, 49, 50].

In this review, we discuss recent discoveries on CNS infiltration by ALL and highlight how they inform future research on CNS disease persistence. We focus on emerging evidence of how ALL persists in the CNS, implicating ALL cell adhesion and metabolic reprogramming in the survival and therapy resistance in this site. Finally, we detail novel therapeutic vulnerabilities and outline targeted therapeutic approaches with the potential to augment current CNS-directed therapy and improve outcomes for ALL patients with CNS disease.

## CNS infiltration by ALL

**Fig. 1:**
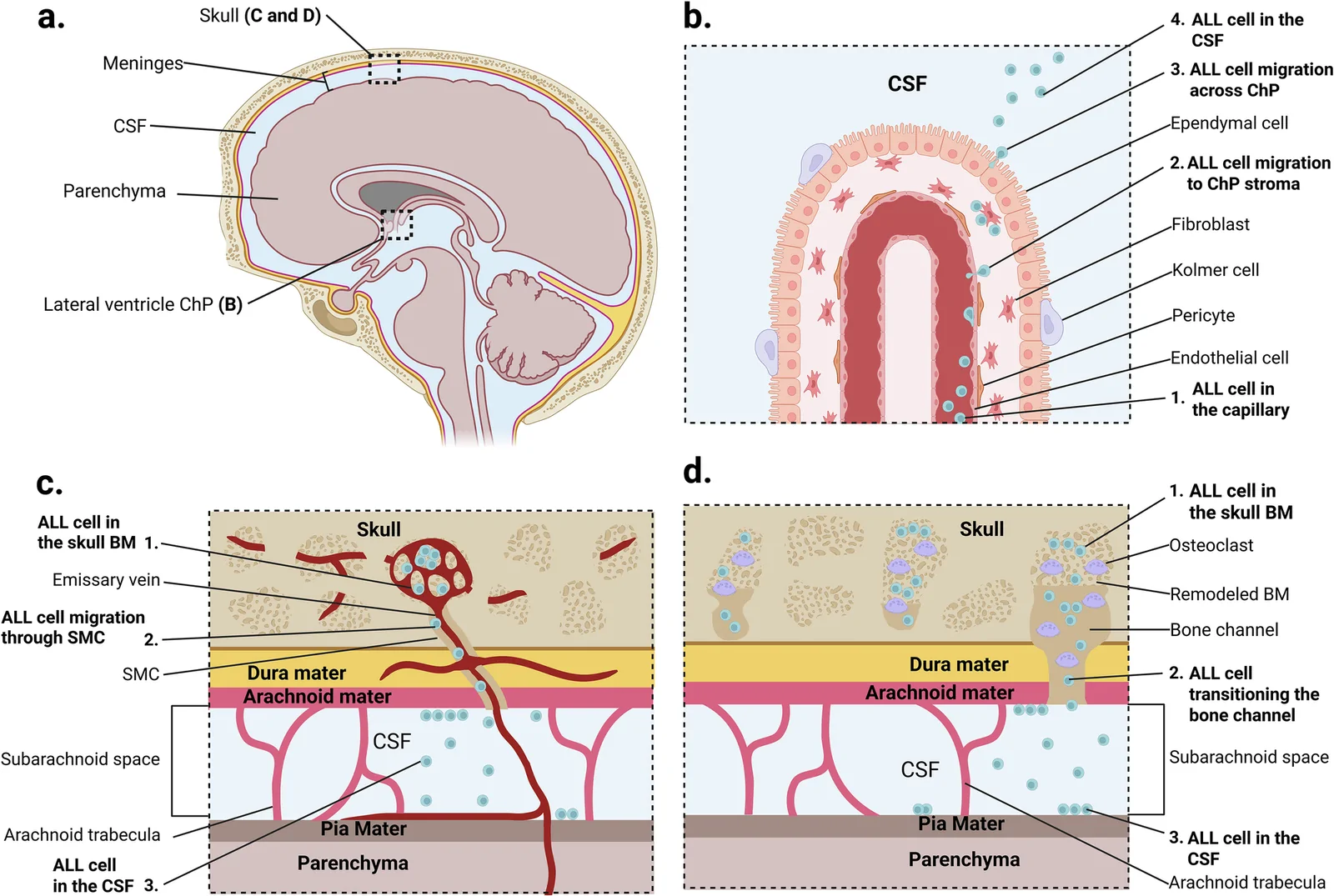
CNS anatomy and proposed modes of CNS infiltration by ALL. **a** A sagittal cross-section of the brain demonstrating relevant CNS anatomy implicated in the CNS infiltration of ALL. **b** A cross-section of the choroid plexus (ChP) comprising capillaries with fenestrated endothelial cells and an outer layer of ependymal cells that encapsulate the ChP stroma. ALL cells travelling through venules of the ChP (1) migrate into the ChP stroma (2) and may cross the ependyma (3) to enter the CSF and invade the leptomeninges (4). **c** A cross-section of the skull BM, emissary veins, skull meninges channels (SMCs), meninges, arachnoid trabecula and parenchyma. ALL cells within the skull BM (1) traverse SMCs (2) and invade the subarachnoid space of the leptomeninges (3). **d** A cross-section of the skull BM, remodelled BM with channels continuous with the subarachnoid space, meninges, arachnoid trabecula and parenchyma. ALL cells within the skull BM (1) induce aberrant activation of osteoclasts that remodel skull BM and form channels continuous with the subarachnoid space that permits invasion of ALL cells (2) into the subarachnoid space of the leptomeninges (3). Created in https://BioRender.com.

CNS involvement in ALL most commonly presents as leukemic meningitis, arising from leukemic infiltration of the meninges, a three-layered membrane that encapsulates the brain and spinal cord (Fig. 1a) [51, 52]. In leukemic meningitis, ALL cells accumulate within the subarachnoid space between the arachnoid and pia mater, collectively termed the leptomeninges [51, 52]. The subarachnoid space is filled with CSF, a nutritionally bleak medium, in physiological conditions, that cushions the CNS and coats ALL cells [51, 53, 54]. In late-stage CNS disease, ALL can also be found in the parenchyma consequent to pia mater degradation [51, 52]. It is largely believed that ALL infiltrates the leptomeninges by migrating across certain CNS barriers, interfaces of the vasculature and CNS, that regulate lymphocyte entry [55]. Despite this, crossing of any one CNS barrier is yet to be recorded in ALL, although preclinical evidence of migration across the blood-CSF barrier at the choroid plexus is accumulating (Fig. 1b) [36, 40, 45, 55]. More recently, avascular routes of ALL trafficking into the CNS from the skull BM have been uncovered, including skull meninges channels and ALL-induced skull BM remodeling [39, 40, 56].

### Infiltration from the vasculature

The blood-CSF barrier modulates CNS immune surveillance under physiological conditions, and the choroid plexus epithelium expresses adhesion molecules, like chemokine ligands CCL19, CCL21, and CXCL12, that license immune entry, suggesting a feasible route for ALL trafficking (Fig. 1b) [57–60]. In physiological conditions, T-cells isolated from the CNS express CCR7 and CXCR4, the receptors for CCL19/CCL21 and CXCL12, respectively, suggesting ALL cells may hijack these signaling axes to gain CNS entry [61, 62]. CCR7 and CXCR4 have been implicated in the migration of ALL cells across CNS barriers in vitro [63, 64]. In vivo, T-ALL can utilise both receptors for CNS infiltration, whereas only CXCR4 has been implicated in B-ALL [40, 56, 65]. Although direct migration across the blood-brain barrier has not been observed in ALL, CCL19-dependent transmigration across human brain microvascular endothelial cell monolayers has been demonstrated in vitro, and infiltrating T-ALL cells are closely associated with CCL19-expressing endothelial cells of brain venules in vivo [63, 65]. Rajakumar et al. also demonstrated a role for CXCR4 in mediating CNS infiltration of B-ALL cells in vivo, contrasting earlier findings by Williams et al. with discrepancies potentially attributable to study design [36, 40].

Expression of pro-inflammatory cytokines, like TNF-α, can induce expression of chemokine ligands on CNS barriers, potentially promoting ALL infiltration, as observed in neuroinflammatory disease [55, 66]. Interestingly, increased CSF TNF-α concentrations were found to precede CNS relapse in a cohort of ALL patients, suggesting that the accumulation of pro-inflammatory mediators may promote leukemic infiltration [67]. TNF-α is elevated in ALL and is higher in the serum of T-ALL compared with B-ALL patients. These findings suggest that heightened inflammatory signaling may promote CNS infiltration and contribute to the increased incidence of CNS disease observed in T-ALL.

### Infiltration from the skull bone marrow

Emerging evidence suggests that ALL can invade the CNS directly from the skull BM [39, 40, 56]. In murine models of ALL, transplanted leukemic cells have been shown to colonize the skull BM early, before overt CNS disease [56]. Following the establishment of ALL in the skull BM, both B-ALL and T-ALL cells can induce extensive BM remodelling, leading to the formation of channels consistent with the subarachnoid space, enabling leptomeningeal invasion (Fig. 1d) [40, 56]. The study by Rajakumar et al. elucidated a mechanism of skull BM remodelling by B-ALL cells, where aberrant osteoclast activation, via the cytokine receptor activator of NF-κB ligand (RANKL), enabled CNS invasion [40, 68]. In addition to the findings in skull BM remodelling, recent evidence suggests B-ALL cells hijack skull-meninges channels implicated in the physiological maintenance of meningeal B-cells [39, 40, 69]. Skull-meninges channels facilitate bi-directional access of the skull BM to the CSF, and are coated in the α6 integrin ligand laminin, enabling leptomeningeal invasion of B-ALL cells in vivo (Fig. 1c) [39, 70, 71]. Whether T-ALL cells also hijack skull-meninges channels after skull BM engraftment is yet to be explored. These findings suggest that the skull BM may serve as a bridging site for ALL, enabling avascular CNS invasion through skull meninges channels and/or skull BM remodelling. Further study is required to determine the relative contribution of skull BM infiltration and vasculature infiltration to the total CNS disease burden.

### Limitations in targeting the mechanisms of CNS infiltration in ALL

Recent advances elucidate convergent CNS infiltration methods between T- and B-ALL phenotypes [40, 56, 64]. Unfortunately, our current understanding of CNS infiltration cannot account for the higher incidence of CNS disease in T-ALL compared with B-ALL, underscoring a significant gap in mechanistic understanding and therapeutic targeting. However, these advances reveal significant mechanistic heterogeneity underlying CNS infiltration in ALL, complicating the development of effective therapeutic strategies to prevent CNS entry. Existing clinical challenges in CNS disease may also complicate the translation of therapeutic strategies that target CNS infiltration in ALL. Most pre-clinical studies targeting the CNS infiltration of ALL initiate treatment after leukemic cell injection, before the establishment of CNS disease, so the relevance to patients with overt CNS involvement remains unclear [36, 40, 56]. Targeting the CNS infiltration of ALL to prevent CNS relapse in patients without overt CNS disease would require biomarkers of CNS leukemia, underscoring the importance of identifying biomarkers of CNS disease in ALL. Recognition of these challenges directs contemporary research to the CNS persistence of ALL cells, to uncover therapeutic vulnerabilities and identify targeted therapeutics that eradicate ALL from this site.

## CNS persistence of ALL

### Adhesion of ALL cells in the CNS

Immune cells isolated from the leptomeninges express cell adhesion molecules such as CXCR4, NCAM1 and ITGAE and are found closely associated with leptomeningeal fibroblasts in physiological conditions [62]. In a pioneering study by Akers et al. it was observed that B-ALL cells in co-culture adhere to cells isolated from the choroid plexus, leptomeninges and parenchyma in vitro [44]. The adherence of T- and B-ALL cells to leptomeningeal cells appears to be crucial to maintain ALL cell viability when cultured in CSF in vitro, suggesting adhesion is necessary for the adaptation of ALL cells to the leptomeningeal microenvironment [50]. Recent research suggests soluble pro-survival factors present in the CSF, like nerve growth factor, can modulate cell survival in B-ALL and may also play a role in CNS disease pathogenesis, warranting further investigation [72, 73]. VLA-4 and LFA-1 appear to be key integrins mediating the cell adhesion of both T- and B-ALL cells to leptomeningeal and choroid plexus epithelial cells [45, 48, 74]. Other integrin interactions, including α6 integrin/laminin (B-ALL) and CD56 (NCAM) (both T- and B-ALL), are yet to be explored in CNS-specific co-culture models of ALL, despite their association with CNS disease and therapy resistance [75–77].

**Fig. 2:**
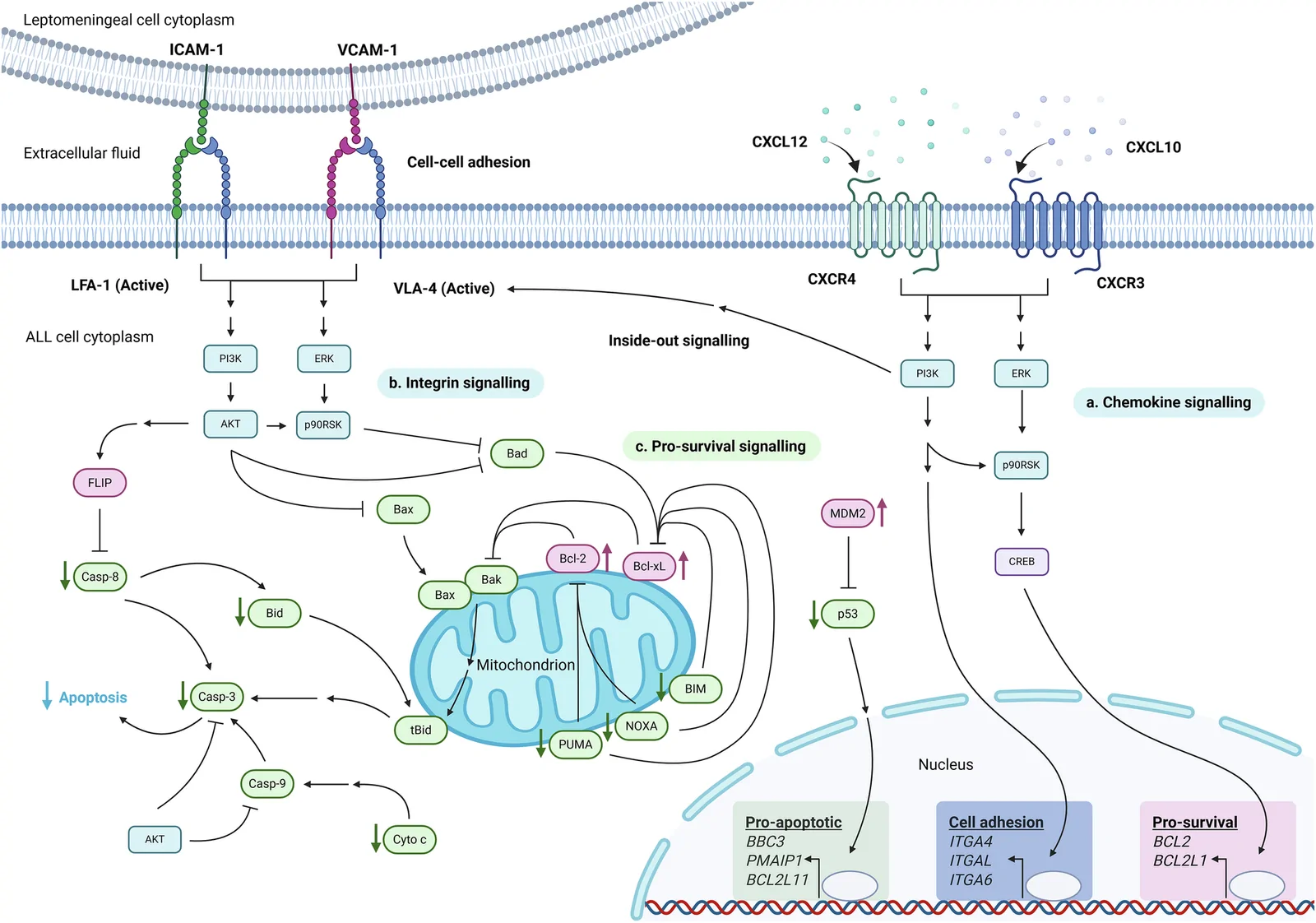
Proposed signaling pathways promoting cell adhesion mediated-drug resistance of ALL in the CNS. The use of two arrows denotes components of a pathway connected through a multi-step process. Blue-boxed proteins represent protein kinases, light purple transcription factors, green pro-apoptotic proteins and pink anti-apoptotic proteins. Colour-matched upward or downward-facing arrows represent increased or decreased pathways, respectively. **a** Chemokine ligands in the leptomeningeal microenvironment, including CXCL10 and CXCL12 can bind to and activate CXCR3 and CXCR4, respectively. CXCR3/CXCR4 activation induces signal transduction through ERK and PI3K that may promote the expression of genes mediating survival and cell adhesion, respectively. Chemokine activation may also promote integrin activation through inside-out signaling. **b** The binding of VLA-4 and LFA-1 to ligands VCAM-1 and ICAM-1 presented by leptomeningeal cells may induce signal transduction through PI3K and ERK. **c** Signal transduction downstream of integrin activation initiates a pro-survival signaling consortium that inhibits apoptosis of ALL cells. CXCL10, C-X-C motif chemokine ligand 10; CXCL12, C-X-C motif chemokine ligand 12; CXCR4, C-X-C motif chemokine receptor 4; CXCR3, C-X-C motif chemokine receptor 3; ERK, extracellular signal-regulated kinase; PI3K, phosphatidylinositol 3-kinase; p90RSK, p90 ribosomal S6 kinase; CREB, cAMP Response Element-Binding Protein; Bcl2, B-cell leukemia/lymphoma 2; BCL2L1, Bcl2 like 1; ITGA4, integrin subunit alpha 4; ITGAL, integrin subunit alpha L; ITGA6, integrin subunit alpha 6; MDM2, murine double minute 2; p53, tumor protein p53; PUMA, p53 upregulated modulator of apoptosis; NOXA, phorbol-12-myristate-13-acetate-induced protein 1; BIM, Bcl-2 interacting mediator of cell death; VLA-4, very late antigen 4; LFA-1, lymphocyte function-associated antigen 1; VCAM-1, vascular cell adhesion molecule 1; ICAM-1, intercellular Adhesion Molecule 1; AKT, Ak train transforming; Bax, Bcl2 associated X; Bad, BCL2 associated agonist of cell death; Bak, Bcl2 antagonist/killer 1; Cyto-c, cytochrome c; Casp-9, caspase-9; Casp-3, caspase-3; Bcl-xL, B-cell lymphoma extra large; Casp-8, caspase-8; BID, BH3 interacting domain death agonist; tBid, truncated Bid. Created in https://BioRender.com.

The expression of VLA-4 (*ITGA4*) and LFA-1 (*ITGAL*) by B-ALL cells and respective ligands VCAM-1 and ICAM-1 by choroid plexus epithelial cells is increased when co-cultured in vitro [45]. Inside-out signaling after chemokine activation is known to regulate integrin activation and expression, and may enhance the adhesion of ALL cells in the CNS (Fig. 2a) [39, 46, 47, 74, 78, 79]. The CXCR4 and CXCR3 ligands, CXCL12 and CXCL10, respectively, are highly concentrated in the CSF [39, 79]. CXCR3 and CXCR4 have been implicated in the adhesion of T-ALL and B-ALL cells, respectively, via VLA-4/V-CAM1 in vitro, suggesting these chemokines may also regulate integrin activity and adhesion of ALL cells in the leptomeningeal microenvironment (Fig. 2a) [46, 47, 74, 80].

Pro-inflammatory signaling also drives the upregulation of multiple classes of cell adhesion molecules, including chemokines and integrins [81]. The pro-inflammatory cytokines IL-8, IL-6 and CCL2 are increased in the supernatant of choroid plexus epithelial cell and B-ALL cell co-cultures in vitro [45]. Additionally, CNS-infiltrating T-ALL cells demonstrate increased expression of genes encoding the pro-inflammatory cytokines IFNG, TNF and IL-27 in vivo [46]. These observations suggest that chemokine and pro-inflammatory signaling may underpin the adhesion of ALL cells in the CNS microenvironment.

#### ALL cell adhesion and therapy resistance in the CNS

The role of cell adhesion-mediated drug resistance (CAM-DR) in ALL chemoresistance within the CNS is becoming increasingly recognized [45, 47, 48]. Both VLA-4 and LFA-1 have been implicated in CAM-DR of both T- and B-ALL in vitro through co-culture with BM stromal cells, with VLA-4 also being implicated in CAM-DR of B-ALL in vivo [82–84]. Recently, Jonart et al. revealed that T- and B-ALL cells co-cultured with leptomeningeal cells could be resensitised to chemotherapy in vitro through isolation and subsequent monoculture [48]. This and other recent work suggest CAM-DR plays a significant role in the chemoresistance of ALL in the CNS via integrins such as VLA-4 and LFA-1 [45, 47, 48].

PI3K/AKT signaling downstream of integrin activation can mediate chemoresistance by altering pro-survival signaling programs(Fig. 2b) [85]. Recent work studying ALL in CNS-specific co-culture elucidates a pro-survival phenotype that could be mediated by PI3K/AKT and underlie the therapy resistance of T- and B-ALL cells in the CNS (Fig. 2c) [48, 86]. AKT can suppress caspase 8 and limit BID activation to resist extrinsic apoptosis, properties of B-ALL cells in CNS-specific co-culture [48, 87]. At the mitochondrial checkpoint of apoptosis, cytochrome C release can be inhibited by AKT, a process reinforced by elevated BCL2 (*BCL2*) and BCL-XL (*BCL2L1*) expression, also observed in T- and B-ALL cells from CNS-specific co-culture [48, 86, 88]. Even after cytochrome C release, AKT activation can rescue cells from apoptosis through the inhibition of caspase 9 [89]. Dysregulated apoptotic control via enhanced BCL2 and BCL-XL signaling and downregulated BIM signaling is implicated in corticosteroid resistance and may blunt dexamethasone responsiveness of ALL in the CNS [90, 91].

Overexpression of *MDM2*, also downstream of AKT, coincides with reduced *TP53* expression in T-ALL cells derived from the CNS of patients, a hallmark of therapy resistance in ALL [86, 92]. Deregulation of p53 in cancer cells promotes survival through downregulation of pro-apoptotic mediators BIM (*BCL2L11*), PUMA (*BBC3*), and NOXA (*PMAIP1*) [93]. Cell cycle arrest is also promoted by reduced p53 levels, observed in T- and B-ALL cells in vitro (CNS-specific co-culture) and B-ALL cells in vivo (CNS-derived), a characteristic associated with resistance to anti-folates, like methotrexate [41, 45, 48, 94, 95]. These findings suggest that adhesion of ALL cells within the CNS microenvironment promotes therapy resistance by enhancing pro-survival signaling and deregulating the p53 pathway.

### Metabolic reprogramming of ALL in the CNS

The leptomeninges is filled with CSF, deprived of fatty acids (FAs), lipids, amino acids and oxygen, amongst several other molecules normally required to support cellular proliferation [42, 43, 53, 54]. These conditions induce metabolic stress, so tumor plasticity has been identified as a critical trait of other cancers metastasising to the leptomeninges [54, 96]. B-ALL cells in the CNS comprise distinct transcriptomic and metabolomic profiles from those in other sites, suggesting tumor plasticity enables metabolic reprogramming and CNS persistence of ALL cells [38, 41–43]. Recent studies implicate hypoxia and lipid deprivation as key microenvironmental factors contributing to altered glucose and lipid metabolism of ALL cells in the CNS [41–43, 97].

#### Glucose metabolism

**Fig. 3:**
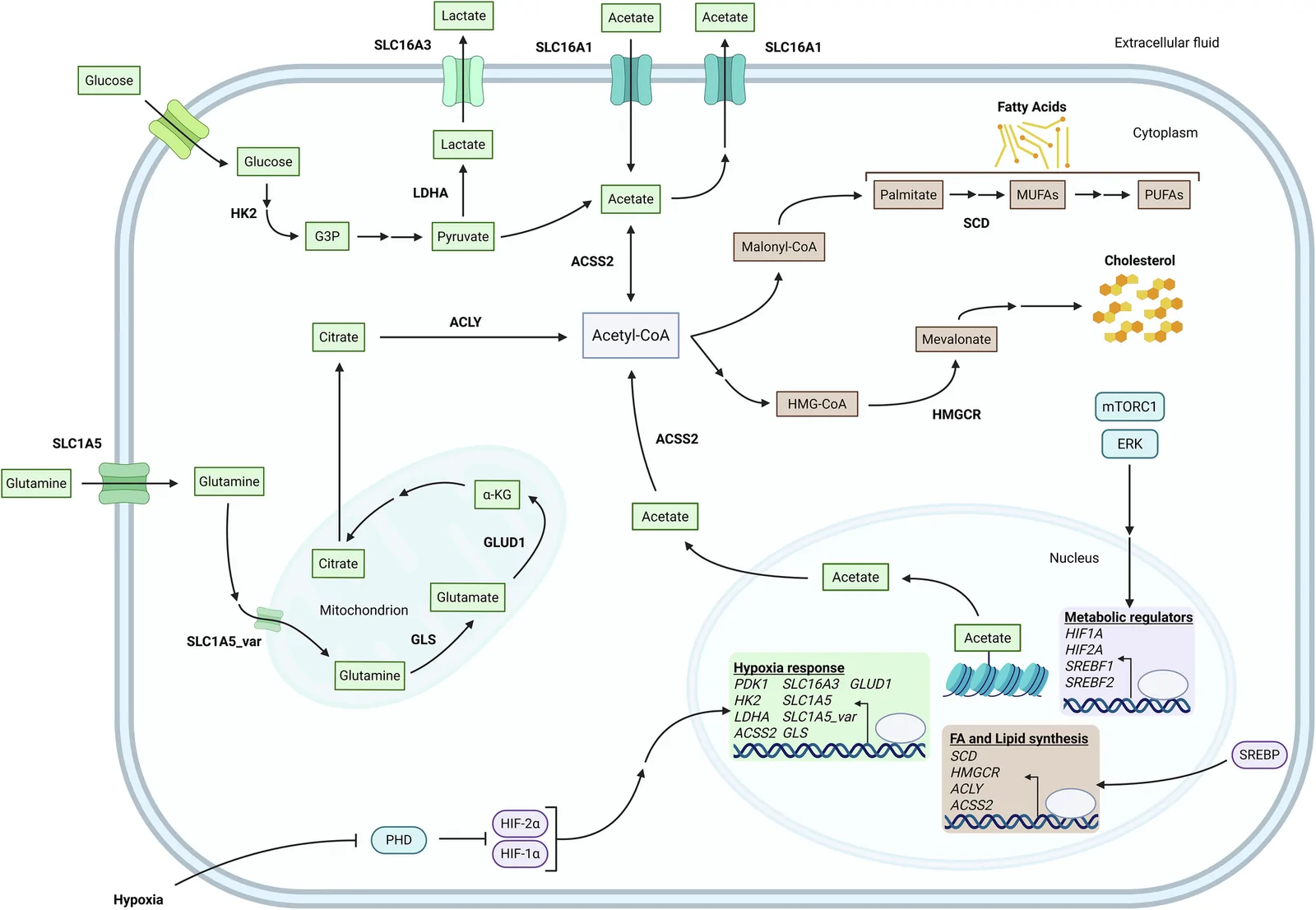
Proposed metabolic pathways active in ALL cells within the CNS microenvironment. The use of two arrows denotes components of a pathway connected through a multi-step process. Hypoxia-inducible factors (HIFs) and sterol regulatory element binding proteins (SREBPs) are transcriptionally regulated downstream of mTORC1 and ERK (Pink). Hypoxia inhibits PHDs, leading to the activation of hypoxia-inducible factor 1-alpha (HIF-1α) and hypoxia-inducible factor 2-alpha (HIF-2α) that stimulate the expression of hypoxia-responsive genes modulating glutamine, glucose, lactate and acetate metabolism (Green). SREBPs stimulate the expression of fatty acid (FA) and lipid synthesis genes that modulate de novo FA and cholesterol synthesis (Brown). mTORC1, mechanistic target of rapamycin complex 1; ERK, extracellular signal-regulated kinase; PHD, prolyl hydroxylase; PDK1, pyruvate dehydrogenase kinase 1; HK2, hexokinase 2; LDHA, lactate dehydrogenase A; ACSS2, acyl-CoA synthetase short-chain family member 2; SLC16A3, solute carrier family 16 member 3; SLC1A5, solute carrier family 1 member 5; SLC1A5_var, solute carrier family 1 member 5 variant; GLS, glutaminase; GLUD1, glutamate dehydrogenase; α-KG, alpha-ketoglutarate; ACLY, ATP citrate lyase; G3P, glyceraldehyde 3-phosphate; SCD, stearoyl-CoA desaturase; HMGCR, 3-hydroxy-3-methylglutaryl-CoA reductase; malonyl-CoA, malonyl coenzyme A; MUFA, monounsaturated fatty acid; PUFA, polyunsaturated fatty acid, HMG-CoA, 3-hydroxy-3-methylglutaryl coenzyme A; Ac, acetate; acetyl-CoA, acetyl coenzyme A. Created in https://BioRender.com.

A hypoxic microenvironment leads to the activation of hypoxia-inducible factors (HIFs), transcription factors that can maintain cancer cell survival by enhancing glycolysis and mitigating oxidative stress (Fig. 3) [98–100]. Hypoxic adaptation of ALL in the CNS has been inferred through upregulation of HIF-1α target genes, including *VEGFA*, *HK2* and *PDK1* and increased levels of glycolytic metabolites of CNS-derived B-ALL cells in vivo [41, 97]. The enhanced glycolysis of CNS-derived B-ALL cells is coupled with decreased mitochondrial oxidative respiration, evidenced through downregulation of relevant gene sets in vivo and a decreased oxygen consumption rate ex vivo [41, 42]. Enhanced glycolysis is also observed in several solid tumor types metastasising to the leptomeninges in vivo, which correlates with elevated CSF lactate, suggesting ALL may also secrete measurable levels of lactate in the CSF (Fig. 3) [41, 54, 101, 102]. These observations suggest that the hypoxic leptomeningeal microenvironment promotes HIF-mediated transcriptional programs, enhancing glycolysis in ALL cells within the CNS.

#### Lipid metabolism

Across several tumor types, including glioblastoma, a lipid-deprived microenvironment leads to the activation of sterol regulatory-element binding proteins (SREBPs), which promote de novo FA and cholesterol synthesis (lipid synthesis), enhancing cancer cell survival by supporting membrane biogenesis and mitigating oxidative stress [103–106].

Transcriptomic analysis reveals B-ALL cells in the CNS upregulate gene sets corresponding with de novo FA and cholesterol synthesis, including SREBP1 (*SREBF1*) and SREBP2 (*SREBF2*), and SREBP-regulated genes *SCD* and *HMGCR* in vivo [42, 43]. Stearoyl-CoA desaturase (SCD) is a key enzyme in de novo FA synthesis, and when overexpressed in B-ALL cells, it enhances de novo FA synthesis in vitro and augments CNS disease in vivo [42, 107]. 3-Hydroxy-3-methylglutaryl-CoA reductase (HMGCR) is the rate-limiting enzyme of cholesterol synthesis, implicated in the survival of ALL cells in lipid-deprived conditions in vitro [43]. Although intracellular levels of cholesterol and the cholesterol precursor mevalonate are increased in CNS-derived B-ALL cells, the role of HMGCR and cholesterol metabolism in this context is yet to be explored in vivo [43]. Interestingly, reduced CSF cholesterol has been associated with active CNS disease in murine models of B-ALL, suggesting CSF cholesterol levels may correlate with CNS disease [43]. Enhanced FA and cholesterol synthesis in hypoxic conditions lead to the accumulation of lipid droplets in clear-cell renal-cell carcinoma cells, yet CNS-derived B-ALL cells have fewer lipid droplets than those derived from the BM in vivo [43, 108, 109]. This finding may reflect a lower nutrient availability for lipid synthesis in the CNS relative to the BM. It could also suggest that ALL cells in the CNS switch to oxidative lipid metabolism under certain conditions, which may underlie the conflicting findings in oxidative respiration in CNS-derived ALL cells by Kato et al. and Vanner et al. warranting further investigation [38, 41].

#### Microenvironment-derived carbon sources for lipid synthesis

Other tumor microenvironments characterised by metabolic stress can induce metabolic reprogramming in cancer cells that drives glutamine and/or acetate metabolism to derive acetyl-CoA for de novo lipid synthesis [110–113]. This is consequent of HIF-mediated inhibition of pyruvate dehydrogenase kinase 1 (PDK1), which prevents glucose-derived acetyl-CoA generation; an essential precursor in both de novo FA and cholesterol synthesis [114]. The metabolic programs of ALL cells in the CNS, guided by HIF and SREBP activation, may also promote glutamine and/or acetate metabolism to fuel lipid synthesis, as observed in other cancers.

Within the CSF, the concentration of glutamine is comparable to the plasma, whereas acetate is scarcely abundant in physiological conditions [115, 116]. Across several solid tumor types, activation of HIF-2α can lead to the increased expression of plasma membrane *SLC1A5* and the mitochondrial membrane variant *SLC1A5_var*, increasing cellular and mitochondrial glutamine import, respectively (Fig. 3) [117, 118]. Furthermore, c-Myc transcriptional activity, induced by HIF-2α, can increase the expression of mitochondrial glutamine-catalysing enzymes GLS and GLUD1, enhancing acetyl-CoA generation [112, 119–121].

Across multiple solid tumor types, it has been demonstrated that acetate can be generated intracellularly via histone deacetylation or de novo from pyruvate and converted to acetyl-CoA by acyl-CoA synthetase short-chain family member 2 (ACSS2) (Fig. 3) [100, 110, 122, 123]. Activation of ACSS2 can also promote epigenomic remodelling that enhances the expression of FA synthesis enzymes, contributing to lipid synthesis [110]. Acetate can be secreted extracellularly via the membrane transporter SLC16A1, measurable in the CSF of patients with leptomeningeal metastasis from lung adenocarcinoma, suggesting ALL cells may also secrete acetate into the CSF [124–126].

It is also conceivable that leptomeningeal cells may provide metabolic support to ALL in the CNS, given that bone marrow mesenchymal cells can support leukemic survival by providing acetate, cysteine and asparagine, representing a considerable gap in CNS disease research [127–129]. These observations suggest that glutamine and/or acetate metabolic reprogramming in ALL cells, potentially reinforced by metabolic support from leptomeningeal cells, may sustain lipid synthesis within the CNS microenvironment.

#### Metabolic reprogramming in the CNS microenvironment: implications for therapy resistance in ALL

In a recent study by Kang et al. it was revealed that culturing ALL cells in CSF induced cell cycle arrest and the integrated stress response (via ATF4), impairing sensitivity to methotrexate [49]. Activation of ATF4 has also been implicated in resistance to L-asparaginase, through induction of asparagine synthesis, and may contribute to therapy resistance of ALL in the CNS [130]. These findings suggest that other metabolic adaptations made by ALL cells in the CNS could contribute to the acquisition of therapy-resistant characteristics, as observed in other cancer contexts.

The alterations to metabolic programs of ALL cells in the CNS, including enhanced HIF signaling and de novo lipid synthesis, are known to contribute to therapy resistance in several tumor types [114, 131]. In breast and colon cancer cells, the activation of HIF-1α increases the expression of numerous drug efflux pumps, including P-gp, BCRP, and MRP1, impairing intracellular drug accumulation [132–134]. In acute myeloid leukemia, the HIF-1α-responsive gene *VEGFA* has been implicated in resistance to cytarabine through enhancing the apoptotic inhibitor protein Survivin [135]. Hypoxia-responsive, AP-1 signaling, recently implicated in the migration of ALL in the CNS in vitro, promotes therapy resistance in breast and liver cancer cells by upregulating the inhibitor of apoptosis, XIAP [136–138]. Across several solid tumour types, enhanced de novo FA synthesis enriches the proportion of intracellular saturated phospholipids, which impairs cell membrane fluidity and subsequent drug penetration [104]. Additionally, enhanced de novo cholesterol synthesis can promote the generation of membrane-bound lipid rafts, which regulate intracellular signaling cascades, involving AKT, Bcl-xL and caspase 3, implicated in the therapy resistance of breast cancer cells [139]. These observations suggest metabolic reprogramming may significantly contribute to the therapy resistance of ALL cells within the CNS, as observed in other cancers, and represents a significant gap in CNS disease research and novel therapeutic discovery for ALL.

## Targeted therapeutic approaches for ALL in the CNS

The development of targeted therapeutics requires a comprehensive molecular understanding of disease. As therapeutic vulnerabilities of ALL in the CNS are uncovered, effective targeted therapies can be developed to clear ALL from this site. Granted, the CNS effectively excludes entry of almost all therapies, including many small-molecule inhibitors and monoclonal antibodies [140]. While novel targeted therapies have transformed the treatment landscape of ALL, their ability to manage disease within the CNS remains uncertain. Blinatumomab, a T-cell-engaging bi-specific antibody, has significantly improved survival for B-ALL but has limited CNS penetration, relying on intrathecal chemotherapy to minimise CNS relapse [141, 142]. CD19/CD22-directed chimeric antigen receptor (CAR) T-cell therapy has recently entered clinical use for B-ALL, but effective CAR’s have yet to be developed for T-ALL [143]. Certain CAR T-cell constructs have measurable CSF penetration, but their contribution to CNS control is yet to be confirmed [144, 145]. These studies underscore CNS disease control as a critical unmet need, reinforcing the urgency for improved CNS-directed therapeutic strategies.

Targeted small-molecule therapeutic approaches have been used to exploit numerous molecular characteristics in ALL cells; the same approach can be applied for ALL in the CNS. Small molecule therapies can be optimised for CNS penetration by reducing their size, polarity, and affinity for efflux transporters, and could enhance the efficacy of current CNS-directed therapy [146–148]. In glioblastoma, the development of CNS-penetrant small-molecule inhibitors has been prioritised; a similar emphasis on CNS pharmacokinetics is warranted when advancing candidate therapies for CNS disease in ALL [149]. Below, we outline promising targeted small-molecule therapies for ALL in the CNS, summarised in Table 1, considering these characteristics.

### Cell adhesion and therapy resistance: therapeutic opportunities

The interactions between leukemic cells and the CNS microenvironment are increasingly recognised as mediators of therapeutic resistance; therefore, cell adhesion molecules have become attractive therapeutic targets. These approaches aim to disrupt distinct cell adhesion modalities, but their therapeutic effect for CNS disease in ALL converges on the same principle: to clear ALL cells from the leptomeninges and re-sensitise them to chemotherapy. The CXCR4 antagonists mavorixafor (AMD070) and plerixafor (AMD3100) reduce the CNS burden of B-ALL and T-ALL, respectively in vivo [47, 56]. Less characterised, the CXCR3 inhibitor AMG-487 enhances survival and reduces clinical symptoms of B-ALL in the CNS in murine models [79].

Targeted therapies antagonising integrins have also shown preclinical promise. The small molecule inhibitor Me6TREN decreases leukemia adhesion by modulating the expression of the VLA-4 ligand, VCAM-1, enhancing the efficacy of cytarabine in the clearance of B-ALL from the CNS in vivo [48]. The PI3Kδ inhibitor GS-649443, despite its poor CNS penetration, reduces the expression of α6 integrin and subsequent CNS infiltration of B-ALL cells in vivo [39, 150].

### Metabolic reprogramming and therapy resistance: Therapeutic opportunities

The role of metabolic reprogramming in therapy resistance of ALL in the CNS is nascent, and few therapeutics have been explored in this context. Central drivers of metabolic adaptation to hypoxia of ALL cells in the CNS may include HIF-1α and HIF-2α [41, 97]. Abemaciclib, a small CDK4/6 inhibitor with measurable CNS penetration, recently demonstrated the ability to inhibit PI3K/mTOR and subsequent HIF-1α expression in vivo [151, 152]. Given that HIF-2α is also regulated by PI3K/mTOR signaling, abemaciclib may also demonstrate activity against HIF-2α [153]. Alternatively, belzutifan, an allosteric inhibitor of HIF-2α, is FDA-approved for CNS hemangioblastomas and may be effective in the treatment of ALL at this site [154]. Downstream of HIF signaling, VEGFA can be antagonised by the monoclonal antibody bevacizumab, which, despite its poor CNS penetration, reduces CNS leukemic burden in PDX models of B-ALL [41, 97]. Additionally, the c-FOS/AP-1 inhibitor T-5224, with unknown CNS pharmacokinetics, attenuates migration of B-ALL cells in a novel in vitro cerebral organoid model; however, its ability to clear ALL cells from the CNS in vivo is yet to be explored [136].

Drivers of de novo FA and cholesterol synthesis in ALL cells within the CNS may also represent attractive therapeutic targets. The tool compound fatostatin inhibits SREBP and demonstrates preclinical efficacy in the treatment of glioblastoma and acute myeloid leukemia [155, 156]. Downstream of SREBPs, the SCD inhibitor SW203668 has unknown CNS pharmacokinetics but effectively decreases the CNS leukemic burden of B-ALL in vivo [42]. Simvastatin can also penetrate the CNS and has been used to target HMGCR, reducing B-ALL viability in vitro*;* however, its lack of efficacy in vivo necessitates alternative therapeutic approaches [43, 157, 158].

### Targeting hallmark signaling effectors PI3K and MEK

ALL subtypes are marked by a collection of genomic alterations that frequently cause aberrant signal transduction through PI3K/AKT/mTOR and Ras/Raf/MEK/ERK [159, 160]. Many of the pathways potentiating survival and therapy resistance of ALL in the CNS are modulated by these same major signaling effectors.

Targeting of both PI3K and MEK has repeatedly demonstrated preclinical efficacy against CNS disease in ALL in vivo [39, 76, 150, 161]. GS-649443, a PI3Kδ inhibitor, and copanlisib, a pan-PI3K inhibitor, reduce CNS leukemic burden and demonstrate synergy with cytarabine, despite their unfavourable CNS pharmacokinetics, in vivo [76, 150]. Copanlisib is currently being investigated in a phase IB/II study for the treatment of primary central nervous system lymphoma (NCT03581942), demonstrating promising initial efficacy [162]. Additionally, the MEK1/2 inhibitor selumetinib, despite its poor CNS penetration, also effectively reduces CNS leukemic burden in PDX models of ALL [161, 163]. A phase I/II study (NCT01089101) is also ongoing using selumetinib for the treatment of recurrent or refractory low-grade glioma, demonstrating promising initial outcomes [164]. Inhibition of PI3K and MEK has demonstrated therapeutic potential in glioblastoma, advancing the development of optimised CNS-penetrant therapies, such as paxalisib and mirdametinib, with excellent brain:plasma ratios in murine models [147, 165]. The phase II study (NCT03522298) revealed that paxalisib enhances outcomes for newly diagnosed glioblastoma [166]. Mirdametinib is currently being investigated in a phase I/II study (NCT04923126), demonstrating early promise for patients with low-grade glioma [167]. These studies frame PI3K and MEK as key therapeutic targets with clinical potential for the treatment of CNS disease in ALL.

## Conclusion

Recent breakthroughs have uncovered mechanisms that enable ALL cells to adapt to the CNS, revealing promising therapeutic vulnerabilities. The diverse mechanisms used by ALL cells to infiltrate the CNS, together with ongoing clinical challenges, complicate the development of effective therapeutic strategies targeting this aspect of the disease. Once ALL has invaded the CNS, the leptomeningeal microenvironment modulates ALL cell biology and may promote therapy resistance through cell adhesion and metabolic reprogramming, representing promising therapeutic vulnerabilities. Considering the immune and pharmacologic privilege of the CNS, these findings frame the CNS as a key therapeutic sanctuary for ALL, impeding the effective clearance of leukemic cells and driving relapse. As diverse elements of the leptomeningeal microenvironment are increasingly implicated in ALL survival and therapy resistance in the CNS, more sophisticated in vitro models with higher fidelity are required to identify effective therapeutic opportunities. Unfortunately, the disproportionate CNS involvement of T-ALL remains unexplained by existing models, and the paucity of studies on T-ALL adhesion and metabolic reprogramming in the CNS exposes key gaps in therapeutic development. Interconnected signaling networks driving adhesion and metabolic reprogramming of ALL in the CNS may pre-empt resistance to single-agent approaches, urging comprehensive disease characterization to identify effective multi-agent treatment strategies. Candidate therapies with favourable CNS pharmacokinetic profiles should be prioritised, given the importance of CNS penetration and drug retention for effective treatment of ALL in this site. Identifying effective targeted therapeutic approaches has the potential to transform CNS-directed therapy in the treatment of ALL, improving patient outcomes and mitigating treatment-related encumbrances.
